# Cross-Cultural Examination of the False Consensus Effect

**DOI:** 10.3389/fpsyg.2019.02747

**Published:** 2019-12-11

**Authors:** Incheol Choi, Oona Cha

**Affiliations:** ^1^Department of Psychology, Seoul National University, Seoul, South Korea; ^2^School of Business Administration, Chung-Ang University, Seoul, South Korea

**Keywords:** false consensus effect, cross-cultural, behavioral choice, European Americans, Koreans

## Abstract

This study explored the cultural differences in the false consensus effect (FCE) between Koreans and European Americans. Two studies adopted a traditional false consensus paradigm and investigated the relative magnitude of the FCE between the two cultures in three different categories of personal choices (Study 1) and behavioral choices involving hypothetical conflict situations (Study 2). The FCE was observed in both the cultures and the effect tended to be stronger among Koreans than European Americans. However, the results from Study 1 also demonstrated that this cultural effect depends on the domain of choices. Cultural implications were discussed.

## Introduction

People tend to perceive their own beliefs, judgments, and attitudes to be more prevalent than what they actually are. Ross et al. (1977) coined the term the *false consensus effect* (FCE) to describe the tendency to “see one’s own behavioral choices and judgments as relatively common and appropriate to existing circumstances while viewing alternative responses as uncommon, deviant, or inappropriate” (p. 280). In the traditional paradigm, participants are given a pair of options, asking to indicate their personal choices and to estimate what proportion of the average others would choose each option. The existence of the FCE is confirmed when the estimates of prevalence for option 1 provided by people who personally chose the same option exceed the estimates provided by those who personally chose the other option. Thus, a false consensus does not necessarily mean that people see their own responses as shared by a *majority* of people. Rather, it is a relative sense of commonness for their own responses, compared to the one perceived by those who provided the alternative responses, which constitutes the false consensus (Ross et al., 1977).

A torrent of research has not only demonstrated the FCE in various settings but also confirmed its empirical robustness (see Mullen et al., 1985; Marks and Miller, 1987, for overview). For example, in a meta-analysis of 155 tests of false consensus hypothesis, the effect was revealed to be highly reliable and of moderate magnitude (average effect size corresponding to a correlation of 0.31; Mullen et al., 1985).

Over 40 years have passed since Ross et al.’s (1977) initial work; however, surprisingly, few studies investigated the potential cultural variations in the FCE. Studies in cultural psychology have furthered our understanding of remarkable cultural differences between Eastern and Western cultures in human cognition, motivation, and behaviors (see Markus and Kitayama, 1991; Nisbett et al., 2001, for overviews); further, they have illuminated crucial mechanisms underlying the various psychological processes. A cultural examination of the FCE will help extend our understanding of the social inference processes underlying the perceived consensus. Although relevant studies are few, existing findings in cultural psychology allow us to speculate with regard to the cultural variations in the FCE. The present research attempted to provide preliminary evidence of a cultural difference in the false consensus between Americans and Koreans in order to fill the gap in the FCE literature as well as to invite further cultural inquiry.

## Culture and Perception of Consensus

Recent cultural studies have revealed considerable cognitive and motivational differences between East Asian and Western cultures. It is now widely understood that diverse cultural fabrics, such as ecologies, philosophical traditions, and economic and social structures, have influenced these cultures into creating different cognitive and motivational lives (e.g., Markus and Kitayama, 1991; Nisbett et al., 2001; Kitayama et al., 2006). For example, collectivistic East Asian cultures were found to have fostered an interdependent idea of the self, whereas individualistic Western cultures fostered an independent idea of the self, and each self-system is found to be associated with distinct cognitive and motivational consequences. But is there any reason to believe there will be cultural differences between East Asians and Westerners in the FCE? Although direct evidence is few, existing findings in cultural psychology allow us to conjecture possible cultural variations.

Attribution research, which was one of the first areas that stirred the interest in cross-cultural variation, seems to suggest East Asians be more sensitive to consensus information compared to Westerners. For example, Cha and Nam (1985) found that Koreans tended to take the base rate or consensus information into account in a greater degree than Americans in explaining an event. They used the McArthur (1976) paradigm, where participants were given a description of an event (e.g., “While dancing, Ralph tripped over Joan’s feet”) and asked to report why the event happened. In this paradigm, providing consensus information (e.g., “almost everyone trips over Joan’s feet”) hardly affected Americans’ explanation of the event; however, Koreans adjusted their explanation by incorporating the consensus information as relevant. Koreans’ relative sensitivity to consensus information is in line with the cultural pattern showing East Asians to be more attentive to the importance of situational information than Westerners when comprehending the world (e.g., see Choi et al., 1999, for a comprehensive review).

The cultural difference in attribution can be traced back in part to how the self is construed in East Asians and Western cultures. In their influential work, Markus and Kitayama (1991) suggested that while Westerners value individuals as unique beings independent of others, East Asians recognize the fundamental interdependence of beings, thus emphasizing the social goal of fitting into socially prescribed roles, standards, and duties and maintaining a harmonious relationship with others. It seems that East Asians strive to get along with others and accommodate themselves to the need of relevant others to a greater extent than Westerners.

Consistent with this notion, not only are East Asians more likely to pay attention to consensus information than Westerners, but they also seem to be *motivated* to seek consensus information. Research suggests that East Asians are more likely to maintain positive self-regard by affiliating with or feeling a sense of connectedness with others (e.g., Heine and Hamamura, 2007); further, the perception of what others think, feel, and behave seems to constitute a crucial basis for positive self-regard for interdependent East Asians. More generally, East Asians are more likely to engage in social comparison than Westerners (Guimond et al., 2007).

Other people’s evaluations and opinions also implicate how good East Asians feel about themselves. For instance, the sense of satisfaction with life in East Asians is heavily affected by subjective norms. Suh et al. (1998) analyzed two large sets of international data containing a total of 61 countries in order to investigate the cultural variation on the basis of life satisfaction. In addition to reporting one’s own global judgment of life satisfaction, participants were also asked to report the normative desirability of life satisfaction: They were asked to imagine being a person who was highly respected and leading a good life and to report life satisfaction from that ideal person’s perspective. The results showed that judgments of life satisfaction were strongly influenced by personal emotional feelings rather than by one’s perception of the norms in individualistic countries. However, in contrast to this Western idea of happiness, people in collectivistic culture were found to base their life satisfaction on norms, i.e., what they think an ideal person in the society might feel, as much as their own emotional reaction.

Cultural studies reviewed so far seem to suggest that East Asians tend to pursue a greater sense of consensus in their lives compared to Westerners. Consistent with the notion, Kim and Markus (1999) found that East Asians and European Americans endorsed considerably different norms regarding uniqueness or “sticking out.” While European Americans showed preference for uniqueness by choosing objects singled out from a group of objects, East Asians showed preference for conformity by choosing the more common object in the sample. Furthermore, it was demonstrated that the cultural theme of uniqueness versus conformity was found to be reproduced and perpetuated through popular media, such as magazine ads, in the respective culture (Kim and Markus, 1999).

The manifestation of the different preference for consensus is not confined to the choice of objects. East Asians also seem to be motivated to see a more similarity between others and themselves compared to Westerners. It was found that typical Americans perceived the self to be more dissimilar to the other than the other is to the self (Holyoak and Gordon, 1983; Srull and Gaelick, 1983), which was understood as an indication that the representation of the self in this culture was more elaborated and distinctive in memory than that of others. However, in a cross-cultural comparison, Kitayama et al. (1990, unpublished, as cited in Markus and Kitayama, 1991) found that this pattern was slightly reversed for Indians: they judged that the self is more similar to the other than the other is to the self.

Ohashi and Yamaguchi (2004) also noted that Japanese seemed to perceive being ordinary or average as safe and satisfying; further, they even make normative self-predictions about their future life events. More specifically, Japanese tend to predict that in comparison with the average person, they are more likely to experience common events rather than rare events. This phenomenon was dubbed as “super-ordinary bias,” since it seemed to be related to viewing oneself as ordinary in an excessive way. Indeed, individuals who viewed themselves as more ordinary tended to show a greater bias.

Similarly, Cho and colleagues reported that collectivistic Koreans were more likely to judge the self to be more similar to others compared to individualistic Koreans (Cho and Kim, 2001; Cho, 2005). In fact, in one of their studies, they provided a list of social issues in Korea and asked participants to estimate what percentage of Koreans would agree with one’s opinions or attitudes on each issue. They found that collectivistic Koreans estimated that more people would share the same opinion or attitude on the given issues or policies compared to individualistic Koreans, which bear an immediate implication for cultural variation in the FCE.

Altogether, cultural findings generally suggest that collectivistic East Asians will demonstrate stronger FCE than individualist Westerners. However, other cultural findings still predict the very opposite. It was also found that East Asians are sensitive to what others do, which results in better knowledge of others. For example, East Asians were found to be rather accurate in estimating the frequency of others’ behaviors (e.g., Ji et al., 2000). Such a result raises the possibility that East Asians may be quite accurate in estimating the prevalence of others’ behaviors, which leads to a less FCE compared to their Western counterparts. Thus, when it comes to the relative size of the FCE between the two cultures, prediction can go in both the directions.

In addition, it is possible that the FCE of Koreans and that of Americans might differ not only in terms of the general size of the effect (e.g., Koreans demonstrate greater FCE than Americans or vice versa) but also in terms of the patterns of domains where they show the effect. In fact, Ross et al. (1977) (Study 2) found that not all domains of choices demonstrated the FCE in the US sample. Examination of a total of 34 items in seven categories of choices revealed the following: 15 of 34 items produced significant FCE beyond the 0.05 level; three (i.e., Political Expectations, Personal Traits/Views, and Personal Problems) of seven categories provided fairly strong and consistent support for the FCE, but only sporadic support for the FCE was provided by Personal Preferences, Personal Characteristics, and Personal Expectations categories, and no significant difference was found for the Personal Activities category (Ross et al., 1977). If this is the case, the domains and choice items that demonstrate FCE may also differ across cultures.

With general negligence of the topic, few studies have examined the cultural variation of the FCE except one recent study by Park (2012). Built on cross-cultural studies, Park (2012) compared the FCE on opinions and attributes items between Koreans and US Americans and found that Koreans exhibited larger FCE than the US Americans, especially on the attribute items. Although this study provides evidence that the FCE differs between Eastern and Western cultures, we still need to establish the cultural pattern as well as to confirm that the results are not confined to the particular type of items that were used in the study.

Acknowledging the gap in the literature, this research aimed to examine the cultural pattern in the FCE. With a limited number of cultural studies available on this topic, we intended to establish the cultural differences by adopting the original setting and paradigm of the FCE from Ross et al. (1977). In two studies, we used the traditional false consensus paradigm and compared the FCE between Koreans and European Americans. Participants were asked to indicate their own choice and to estimate the percentage of their peers who would choose each option. The FCE is confirmed when the consensus estimates, provided by those who endorsed it, exceed the same estimate by those who endorsed the other. Furthermore, the difference between these two estimates can be conceptualized as the magnitude of the FCE.

In Study 1, we first attempted to explore the cultural pattern of the FCE using different types of choices. As reviewed earlier, the FCE was rather pronounced when it involved choices regarding Political Expectations, Personal Traits and Views, and Personal Problems categories; however, it was rather weak for others in the US sample. Since the existence of the FCE depends on the domains, it is possible that the cultural difference can be also evidenced in the types of choice sets that demonstrate the FCE: types of choice category demonstrating strong FCE might reflect different cultural meanings and importance that is specific to the culture. Thus, this possibility was explored in Study 1 using three different categories of choices.

In Study 2, we compared the FCE regarding behavioral choices between Koreans and US Americans, using two hypothetical conflict situations that were adopted from Ross et al. (1977). First, we examined the cultural difference by comparing the magnitude of the FCE on behavioral choices. In addition, we conducted accuracy analyses on the estimation of consensus in order to see whether there is a difference in the estimation processes between cultures and whether this difference can contribute to the cultural pattern in the FCE.

## Study 1

Previously, Ross et al. (1977) reported that support for the FCE was varied by the types of choices. The original questionnaire items by Ross et al. (1977) were organized into seven different categories: Personal Traits and Views, Personal Preferences, Personal Characteristics, Personal Activities, Personal Problems, Personal Expectations, and Political Expectations. Among these categories, only three categories, i.e., Political Expectations, followed by Personal Problems and Personal Traits/Views, demonstrated fairly strong and consistent FCE; rather weak and sporadic effects were found in Personal Preferences, Characteristics, and Expectations, and no effect was noted in the Personal Activities category (Ross et al., 1977). In this study, we examined the existence of the FCE in three categories of choices that demonstrated strong and reliable effects, i.e., Political Expectations, Personal Problems, and Personal Traits/Views, and the cultural pattern of the FCE in different domains.

### Method

#### Participants

Seventy US undergraduates who identified themselves as European Americans and 53 Korean undergraduates at a large university in the respective country participated in this study as a partial fulfillment of a course requirement.

#### Procedure

The study complied with the Declaration of Helsinki. An ethics approval was not required for the study as per applicable institutional and national guidelines and regulations. Participation was voluntary, and participants were informed that they could quit at any time. Written and informed consent was obtained from all participants.

The study material was adopted from Ross et al.’s (1977) classic paradigm with partial modifications to reflect the time and socio-cultural changes. The questionnaire included various binary choice items with mutually exclusive and exhaustive options. For each item, participants were asked to indicate their own choice of answer that applies to them the most and to estimate the proportion of their peers who would choose each option from 0 to 100. The percentage estimates for the two options in each question should add up to 100.

In the original study by Ross et al. (1977), three of seven categories, i.e., Political Expectations, Personal Problems, and Personal Traits/Views, demonstrated fairly strong and consistent FCE; other categories yielded mixed results. Therefore, the three categories that demonstrated a stable effect in the original study were employed for the cross-cultural comparison. There were five items each for Personal Problems and Personal Traits/Views and four items in the Political Expectations category in the original versions. Since some items were culture-bound or were outdated, some of the items were modified or excluded from the final analyses. For example, items such as “woman in Supreme Court within a decade” and “Discovery of extraterrestrial life within next 20 years” in Political Expectation category were modified into “woman president within a decade” and “Discovery of extraterrestrial life within next 20 years,” respectively. In addition, due to the unique sociopolitical circumstances that Korea has suffered, people who announce themselves as politically left of the center are still considered as extremists and stigmatized to a certain degree. Therefore, “politically left of center” in Personal Traits/Views were excluded from the analyses. As a result, a total of 13 items were analyzed and reported here—four items in the Political Expectations, the Personal Traits/Views, and five items in the Personal Problems (see Table 1 for complete list). The materials were translated and back-translated for Koreans, and the participants completed the questionnaire in their own languages.

**Table 1:** Estimated percentages of “college students in general” who would choose option 1.

Items	European Americans				South Koreans			
Option 1 (option 2)	Those who chose option 1	Those who chose option 2	*t*	Cohen’s *d*	Those who chose option 1	Those who chose option 2	*t*	Cohen’s *d*
**Political expectations**								
Female president within a decade? yes (no)	45.67	21.98	4.29^***^	1.03	55.60	17.15	4.80^***^	2.16
Poverty problem reduced in the next 20 years? yes (no)	51.81	35.92	3.22^**^	0.85	67.54	21.63	8.12^***^	2.47
Discovery of extraterrestrial life within next 20 years? yes (no)	42.62	28.76	2.79^**^	0.66	51.21	23.94	4.81^***^	1.29
Nuclear warfare in the next 20 years? yes (no)	53.33	31.20	4.76^***^	1.14	49.29	21.83	4.25^***^	1.36
**Personal problems**								
Think about dying? yes (no)	53.86	24.18	5.74^***^	1.33	80.20	57.14	3.16^**^	1.18
Hard to make friends? yes (no)	34.46	29.13	1.26	0.34	53.57	33.00	3.28^**^	0.95
Difficulty controlling temper? yes (no)	45.80	37.85	1.28	0.42	57.37	35.76	3.42^**^	1.04
Frequently depressed? yes (no)	41.25	28.25	2.56^*^	0.68	58.46	33.67	6.05^***^	1.66
Emotional needs satisfied? yes (no)	55.96	43.65	2.23^*^	0.58	52.17	37.17	3.41^**^	0.94
**Personal traits/views**								
Shy (not shy)	42.27	36.81	1.35	0.36	49.16	46.45	< 1	0.15
Optimistic (not)	64.54	47.00	2.54^*^	1.23	59.29	37.00	5.03^***^	1.52
Competitive (not)	74.04	64.20	2.26^*^	0.56	62.86	65.22	< 1	0.11
Supporter of women’s liberty (not)	72.82	26.67	4.38^***^	3.45	49.62	35.62	2.50^*^	0.85

* p < 0.05;

** p < 0.01;

*** *p < 0.001*.

### Results and Discussion

First, participants’ responses were examined separately by culture in order to confirm the existence of the FCE within each culture. Then, the data were subjected to a 2 × 2 (culture × choice) analysis.

#### Within Each Culture

The data were analyzed within each culture in order to verify the general existence of the FCE. Following Ross et al.’s (1977) analysis, we examined the number of items that demonstrated a significant FCE in each category. Of the 13 items, Americans showed FCE on 10 items and Koreans showed the effect on 11 items at a 0.05 level (see Table 1). Our results from Americans were fairly reminiscent of the results from Ross et al. (1977). Consistent with the previous finding, consistent FCEs were observed in the domain of Political Expectations (4 of 4 items), followed by Personal Problems (3 of 5 items) and Personal Traits/Views (3 of 4 items).

Although European American data resembled the original findings in Ross et al.’s (1977) study, there were notable differences as well as similarities in the Korean results. Similar to the European Americans, Koreans showed a robust FCE in Political Expectations (4 of 4 items) and Personal Problems (5 of 5 items). However, unlike European Americans, Koreans showed rather sporadic support for the FCE in Personal Traits/Views items (2 of 4 items).

#### Between Cultures

The mean consensus estimates for option 1 of each category were subjected to a 2 × 2 (Culture: Korean vs. American × Rater’s Choice: Option 1 vs. Option 2) ANOVA. First, there were significant main effects of Rater’s Choice [Political Expectations: *F*(1, 483) = 177.77, *p* < 0.001, $\eta_{p}^{2}$ = 0.269; Personal Problems: *F*(1, 605) = 209.91, *p* < 0.001, $\eta_{p}^{2}$ = 0.258; Personal Traits/Views: *F*(1, 486) = 57.99, *p* < 0.001, $\eta_{p}^{2}$ = 0.150]: the estimates of prevalence for option 1 by those who personally chose the same option exceed the estimates provided by those who chose the other. When the cultures were combined, the data generally confirmed the FCE across different domains.

There was a significant main effect of culture found in Personal Problems and Personal Traits/Views [Political Expectations: *F*(1, 483) < 1, ns, $\eta_{p}^{2}$ = 0.000; Personal Problems: *F*(1, 605) = 29.77, *p* < 0.001, $\eta_{p}^{2}$ = 0.047; Personal Traits/Views: *F*(1, 486) = 5.16, *p* = 0.02; $\eta_{p}^{2}$ = 0.040]: Koreans’ estimates exceed Americans’ estimates in Personal Problems items, while Americans’ estimates exceed Koreans’ estimates for items in Personal Traits/Views. However, these effects were constrained by Culture × Choice interactions.

Significant interaction effects were found in Political Expectations [*F*(1, 483) = 16.42, *p* < 0.001, $\eta_{p}^{2}$ = 0.033], Personal Problems [*F*(1, 605) = 6.51, *p* = 0.01, $\eta_{p}^{2}$ = 0.011], and Personal Traits/Views [*F*(1, 486) = 12.36, *p* < 0.001, $\eta_{p}^{2}$ = 0.009]. As for Political Expectations and Personal Problems, Koreans were found to show greater FCE than Americans. However, the pattern was reversed for Personal Traits/Views items.

In sum, the result confirmed the general existence of the FCE in both cultures. In addition, the overall pattern seems to suggest that FCE is more pronounced among Koreans than Americans. However, the result also demonstrated some irregularities: Koreans demonstrated less FCE than Americans on Personal Traits/Views.

The cultural pattern we found on Personal Traits/Views items was rather unexpected. We speculated that Koreans demonstrated less FCE compared to US Americans in this category due to a less clear sense of their self-concept. According to the cultural findings, collectivistic and interdependent East Asian self is qualitatively different from the highly individualized independent North American self (see Heine, 2001 for a review): the North American self is characterized by abstract traits and abilities consistent across different situations; however, the East Asian self is largely a relational phenomenon (Markus and Kitayama, 1991; Kanagawa et al., 2001), and an individual’s relationships and roles take precedence over the internalized attributes (Heine, 2001). For example, Americans’ self-description tended to use pure psychological attributes and showed little change across situations, whereas Japanese and Koreans’ self-descriptions involved less use of abstract attributes and varied significantly across situations (Bond and Cheung, 1983; Kanagawa et al., 2001; Suh, 2002). Similarly, it was found that compared to European Americans, Asian Americans were less consistent in their self-descriptions across relationship contexts (English and Chen, 2007) and self-concept clarity or the consistency of the self across situations and time was found to have a strong correlation with self-esteem and well-being for Americans and Canadians, which was not the case for East Asians (Campbell et al., 1996; Suh, 2002). The Personal Traits/Views category that we used in this study was mostly composed of trait items; hence, it was possible that Koreans for whom changing roles depending on situational factors took precedence over stable personal traits would make a less strong estimation on these items.

Based on this reasoning, we examined whether Koreans’ estimates for Personal Traits/Views items were significantly different from an uncertain guess of 50. We have conducted a one-sample *t* test against the value of 50 on the estimates provided by Koreans and US Americans. The results revealed that most of the estimates provided by Koreans for Personal Traits/Views were not significantly different from 50 [*t*(269) = −1.39, n.s.], whereas those provided by European Americans were significantly larger than 50 [*t*(343) = 8.90, *p* < 0.001]^1^. This pattern did not show up in Political Expectations [Koreans: *t*(215) = −14.24, *p* < 0.001; Americans: *t*(274) = −10.41, *p* < 0.001]^2^ or in Personal Problems [Koreans: *t*(215) = −4.57, *p* < 0.001; Americans: *t*(274) = −10.13, *p* < 0.001]^3^.

Overall, the cross-cultural examination of the FCE on different types of choice set demonstrated some cultural variations and similarities. Both Americans and Koreans showed reliable FCEs in three categories of choice that were first evidenced in Ross et al.’s (1977) original study. However, the cultural pattern of the FCE was different depending on the choice sets: Koreans generally demonstrated greater FCE than Americans in choices regarding Political Expectations and Personal Problems. However, this cultural pattern was reversed for Personal Traits/Views, resulting in Americans showing greater FCE. An additional analysis revealed that this was due to the fact that Koreans did not make a strong estimation for Personal Traits compared to Americans, which is consistent with the idea that unlike Americans, Koreans do not count on a strong sense of personal traits. This result suggests that the cultural pattern of the FCE informs us with different cultural meanings that each domain of choice has for its respective culture.

Although we have some initial evidence showing a cultural difference in FCE between Koreans and Americans, it seems that a list of binary choices without proper context complicates the interpretation of the cultural pattern. Therefore, in the next study, we used hypothetical situations that involved an expression of opinions and more realistic behavioral choices.

## Study 2

This study adopted a choice task involving hypothetical situations from Ross et al. (1977). Participants from Korea and the United States were given descriptions of hypothetical situations and were asked to make choices between two options. Two stories that contained decisions that ordinary college students may encounter in both cultures were selected, i.e., the Term Paper story and the Supermarket story. After reading each story, participants were asked to indicate their own choices and provide percentage estimates of people who would choose each option using a scale ranging from 0 to 100.

We examined whether the FCE could be evidenced in the respective culture and whether the FCE was different across cultures. The existence of the false consensus is determined when the consensus estimates for an option provided by people who personally endorsed it surpass the same estimate provided by people who did not choose the option. Furthermore, the difference between these two estimates can be conceptualized as the magnitude of the FCE, and this was compared between the two cultures. Unlike the original study, we also conducted an accuracy analysis by subtracting the actual consensus in the sample in order to see which process was responsible for the patterns of FCE.

### Method

#### Participants

A total of 57 American undergraduates identifying themselves as European Americans and 53 Korean undergraduates at a large university in the respective country participated in this study as a partial fulfillment of course requirements. The data of the participants who did not complete the questionnaire were excluded from the following analyses. Responses from 52 European Americans and 49 Koreans were subject to final analyses.

#### Materials

Materials and general procedures were adopted from Study 1 of Ross et al. (1977). Participants were given brief descriptions of hypothetical conflict situations that eventually asked for a behavioral choice between two options. From the original set of situations, two stories were selected: The Term Paper story and the Supermarket story. These stories were selected because they contained situations and decisions that ordinary college students may encounter in both the cultures.

In the Term Paper story, students faced a situation where they have to decide to write a final paper individually or as a team for a course. The whole story read as follows:

You arrive for the first day of class in a course in your major area of study. The professor says that the grade in your course will depend on a paper due the final day of the course. He gives the class the option of two alternatives upon which they must vote. They can either do papers individually in the normal way or they can work in teams of three persons who will submit a single paper between them. You are informed that he will still give out the same number of A’s, B’s and C’s, etc., but that in the first case, every student will be graded individually while the second case, all three students who work together get the same grade.

In the Supermarket story, students were given a situation where people from a supermarket chain unwittingly filmed their comments about the supermarket and were asking participants to sign a release to use the film for a TV commercial. The story continued as follows:

As you are leaving your neighborhood supermarket, a man in a business suit asks you whether you like shopping in that store. You reply quite honestly that you do like shopping there and indicate that in addition to being close to your home, the supermarket seems to have very good meat and produce at reasonably low prices. The man then reveals that a videotape crew has filmed your comments and asks you to sign a release allowing them to use the unedited film for a TV commercial that the supermarket chain is preparing.

After reading each story, participants estimated the proportion of peers who would agree to choose each option (Paper: group vs. individual papers; Supermarket: agreeing vs. refusing to sign the release) and indicated their own choices. The materials were translated and back-translated into Korean for the Korean participants.

#### Procedures

The study complied with the Declaration of Helsinki. An ethics approval was not required for the study as per applicable institutional and national guidelines and regulations. Participation was voluntary, and participants were informed that they could quit at any time. Written and informed consent was obtained from all participants.

On arrival at the laboratory, participants were presented with a packet containing the descriptions of two hypothetical situations. After reading each story, they were asked to estimate the percentages of their peers who would choose option 1 or 2. Participants were also asked to indicate which behavioral alternative they would choose personally. After completing the questionnaire, participants were fully debriefed and dismissed.

### Results and Discussion

The data were analyzed in two ways. First, we adopted Ross et al.’s (1977) original procedure comparing the estimated consensus for an option between people who endorsed the very option and people who did not. This allows us to confirm the existence of the FCE in Koreans and European Americans and to explore whether there is any cultural difference in the magnitude of the effect between the two cultures. Second, we also attempted to investigate the accuracy of people’s estimation in order to see whether there is a cultural difference in the estimation process.

#### Estimated Consensus

According to Ross et al. (1977), the existence of the FCE can be identified by showing that the estimates of consensus for option 1 provided by those who personally chose option 1 are greater than the same estimates provided by those who personally chose the other option, i.e., option 2. Thus, consensus estimates for option 1 (voting for group paper; signing the release) provided by people who personally chose options 1 and 2 were compared through independent sample *t* tests. The analysis of the perceived consensus in each culture shows that FCE is quite prevalent (see Table 2). Regardless of the type of stories, both Americans and Koreans who chose option 1 estimated that their peers would choose the same option significantly more compared to those who chose option 2 [Koreans: *t*(47) = 6.56, *p* < 0.001, Cohen’s *d* = 1.89 for the Paper story; *t*(47) = 10.54, *p* < 0.001, Cohen’s *d* = 3.02 for the Supermarket story; European Americans: *t*(49.91) = 2.91, *p* < 0.01, Cohen’s *d* = 0.77 for the Paper story; *t*(50) = 2.39, *p* < 0.05, Cohen’s *d* = 0.69 for the Supermarket story].

**Table 2:** Perceived consensus within each culture: estimated percentage of people who would choose option 1 provided by the raters who chose either option 1 or option 2.

Culture	Story	Rater’s own choice	*n* (%)	Perceived consensus on option 1	Actual percentage of peers who chose option 1	*t* (*p*-value)
Koreans	Term paper story	Group (option 1)	31 (63.27)	67.10	62.50	6.56^***^
		Individual	18 (36.73)	38.33	64.58	(0.00)
	Supermarket story	Sign (option 1)	24 (48.98)	71.46	47.92	10.54^***^
		Refuse to Sign	25 (51.02)	32.20	50.00	(0.00)
European Americans	Term paper story	Group (option 1)	19 (35.29)	69.21	35.29	2.91^**^
		Individual	33 (64.71)	55.06	37.25	(0.01)
	Supermarket story	Sign (option 1)	34 (65.38)	78.59	64.71	2.39^*^
		Refuse to Sign	18 (34.62)	69.72	66.67	(0.02)

*Actual percentage of peers who chose option 1 was calculated differently depending on their choices. E.g., actual percentage of peers who chose option 1 for Term Paper story was 30/48 for Koreans who chose option 1 themselves but 31/48 for those who chose option 2*.

* p < 0.05;

** p < 0.01;

*** *p < 0.001*.

In order to compare the FCE cross-culturally, the estimated percentages of people who would choose option 1 were subjected to a 2 × 2 (Culture: Korean vs. European American × Rater’s Choice: Option 1 vs. Option 2) ANOVA. For each story, there was a significant main effect of Rater’s Choice, which confirms the general tendency of FCE across cultures [Paper: *F*(1, 97) = 35.42, *p* < 0.001, $\eta_{p}^{2}$ = 0.292; Supermarket: *F*(1, 97) = 83.77, *p* < 0.001, $\eta_{p}^{2}$ = 0.463]. In addition, there was a significant main effect of Culture [Paper: *F*(1, 97) = 6.83, *p* = 0.01, $\eta_{p}^{2}$ = 0.066; Supermarket: *F*(1, 97) = 72.12, *p* < 0.001, $\eta_{p}^{2}$ = 0.426]. This suggests that Koreans made lower estimations for the percentage of people who would prefer option 1 than Americans.

**Figure 1:**
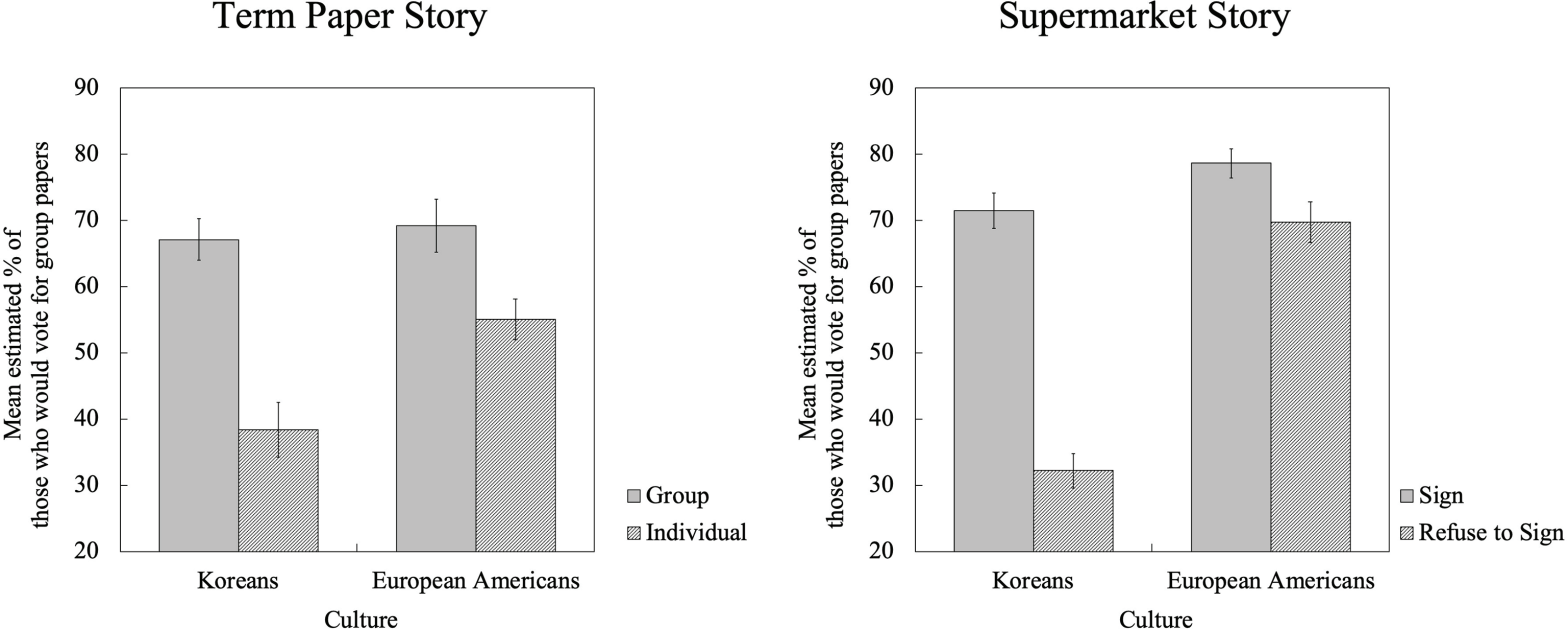
Perceived consensus: Mean estimated percentage of people who would choose option 1 (Term Paper story: Voting for group papers; Supermarket story: Signing the release) by culture and raters’ personal choices.

But more importantly, this effect was constrained by a significant interaction between Culture and Rater’s Choice [Paper: *F*(1, 97) = 4.11, *p* < 0.05, $\eta_{p}^{2}$ = 0.041; Supermarket: *F*(1, 97) = 33.41, *p* < 0.001, $\eta_{p}^{2}$ = 0.256]. The interaction was largely driven by the difference in the perceived consensus on option 1 depending on people’s personal choices: The differences in consensus estimates for option 1 provided by those who personally endorsed options 1 and 2 were greater among Koreans compared to European Americans. For example, in the Supermarket story, Koreans who personally preferred to sign the release (option 1) estimated that 71.46% of fellow college students would choose the same option, whereas those who preferred not to sign it (option 2) thought only 32.20% of them would choose to sign the release. The difference in the estimates of Koreans for option 1 was 39.26%. However, in the same story, European Americans who chose to sign the release (option 1) estimated that the percentage of people who would choose the same to be 78.59%, and those who personally preferred not to sign the release (option 2) estimated that 69.72% of college students would sign it. Thus, the difference in the estimates of European Americans for option 1 was only 8.87%, which is smaller than that of Koreans. The similar pattern applied to the Term Paper story as well. Because the difference in consensus estimates can serve as an indicator for the size of the FCE, this pattern suggests that Koreans show larger FCE than European Americans (see Figure 1).

In sum, the FCE was observed to be a robust phenomenon in this traditional paradigm using hypothetical situations. People from both cultures demonstrated the FCE. However, the comparison revealed some cultural variations as well: Koreans seemed to exhibit greater FCE than European Americans.

#### Accuracy

Although the previous analysis revealed the existence of the cultural variation, it does not allow us to understand what had happened in the estimation. Another way to look at these data is to see whether people’s estimation was accurate by comparing people’s estimated endorsement of an option with the actual percentage consensus in the sample. Especially, some researchers even have suggested that East Asians might be more keen to consensus information and might show less false consensus; thus, the additional accuracy analyses might shed some light on this perspective.

In order to analyze the accuracy of estimation, we first calculated the difference scores between the estimated consensus and actual consensus. We subtracted the actual percentage of peers who chose option 1 in each sample (see Table 2) from the estimated consensus provided for option 1.

The accuracy of estimation can be examined in two ways: First, analysis on the absolute value of the difference scores can determine whether the estimation was deviant from the actual consensus or not; second, analysis on the raw difference score can provide information regarding the direction of the deviation, i.e., whether participants were overestimating or underestimating. Results from these two analyses were presented in order.

First, we used the absolute value of the difference in order to see whether accurate estimation of Koreans or Americans has contributed to the cultural pattern. Scores close to zero indicate accurate judgments, and any deviation from zero indicates an inaccurate estimation. The 2 × 2 (Culture: Korean vs. European American × Raters’ Choice: Option 1 vs. Option 2) ANOVA on the absolute score revealed slightly different results depending on the type of story.

As for the Term Paper story, there was a significant main effect of Culture and a Culture × Choice interaction [Culture: *F*(1, 97) = 16.73, *p* < 0.001, $\eta_{p}^{2}$ = 0.147; Culture × Choice: *F*(1, 97) = 21.76, *p* < 0.001, $\eta_{p}^{2}$ = 0.183]. In this story, Koreans were generally more accurate than European Americans (Koreans: *M* = 17.36, SD = 13.36; European Americans: *M* = 28.51, SD = 13.50), but this pattern was further qualified by Culture × Choice interaction: Koreans who chose option 1 were more accurate (*M* = 11.85, SD = 8.04) than those who chose option 2 (*M* = 26.85, SD = 15.48), whereas European Americans who chose option 2 tended to be more accurate (*M* = 25.40, SD = 13.31) than those who chose option 1 (*M* = 33.92, SD = 12.36).

As for the Supermarket story, there were significant main effects of Choice and Culture, but no interaction effect [Choice: *F*(1, 97) = 6.56, *p* < 0.05, $\eta_{p}^{2}$ = 0.063; Culture: *F*(1, 97) = 13.08, *p* < 0.01, $\eta_{p}^{2}$ = 0.119]. Regardless of culture, estimation on option 2 (*M* = 15.66; SD = 11.15) was more accurate than estimation on option 1: (*M* = 19.65; SD = 10.14) in this story. Regarding the main effect of Culture, European Americans were found to be more accurate than Koreans (Koreans: *M* = 21.34, SD = 11.97; European Americans: *M* = 14.76, SD = 8.30).

The results show that neither Koreans nor Americans were, in general, more accurate than the other, as some researchers have suggested. Rather, their accuracy depended on the type of story. Furthermore, even in the case where Koreans were found to be more accurate than Americans, their accuracy did not lead them to show less FCE. Therefore, the hypothesis stating that Koreans should show less FCE than Americans because they have more accurate knowledge of others does not seem to hold in our study. The accuracy of estimation in an absolute sense does not provide a meaningful explanation for the cultural pattern we found.

The next analysis involved the raw difference scores. Scores close to zero indicate accurate judgments, and any deviation from zero indicates an inaccurate estimation with directions: Positive scores indicate overestimation, whereas negative scores indicate underestimation. The difference scores from each story were subjected to a 2 × 2 (Culture: Korean vs. European American × Raters’ Choice: Option 1 vs. Option 2) ANOVA. The mean difference scores in each condition and the results of the significance tests comparing the difference scores against a value of zero are presented in Table 3.

**Table 3:** Accuracy of estimation: Actual percentage of peers who chose option 1 subtracted from the estimated percentage of people who would choose option 1 with significance tests against zero.

Culture	Story	Rater’s own choice	Perceived consensus – Actual percentage	*t*
Koreans	Term paper	Group	4.60	1.87
		Individual	−26.25	−6.73^***^
	Supermarket	Sign	23.54	9.67^***^
		Refuse to Sign	−17.80	−6.36^***^
European Americans	Term paper	Group	33.92	11.96^***^
		Individual	17.80	4.51^***^
	Supermarket	Sign	13.88	6.48^***^
		Refuse to Sign	3.05	0.98

*** *p < 0.001*.

First, there was a significant main effect of Choice [Term Paper: *F*(1, 97) = 42.41, *p* < 0.001, $\eta_{p}^{2}$ = 0.304; Supermarket: *F*(1, 97) = 98.42, *p* < 0.001, $\eta_{p}^{2}$ = 0.504]. Regardless of culture, the degree of overestimation of the consensus was greater for their own choice than for the other choice in the Term Paper story (Option 1: *M* = 15.74, SD = 19.44; Option 2: *M* = 2.26, SD = 29.58) and in the Supermarket story (Option 1: *M* = 17.88, SD = 13.06; Option 2: *M* = −9.07, SD = 17.06), which is consistent with the notion of the FCE. The analysis also revealed a main effect of Culture [Paper: *F*(1, 97) = 103.58, *p* < 0.001, $\eta_{p}^{2}$ = 0.516; Supermarket: *F*(1, 97) = 4.53, *p* < 0.05, $\eta_{p}^{2}$ = 0.045]. The pattern suggested that regardless of their personal choices, consensus overestimation in general was stronger for Americans than for Koreans in the Term Paper story (Koreans: *M* = −6.73, SD = 20.98; European Americans: *M* = 23.70, SD = 20.95) and in the Supermarket story (Koreans: *M* = 2.45, SD = 24.54; European Americans: *M* = 10.13, SD = 13.64).

**Figure 2:**
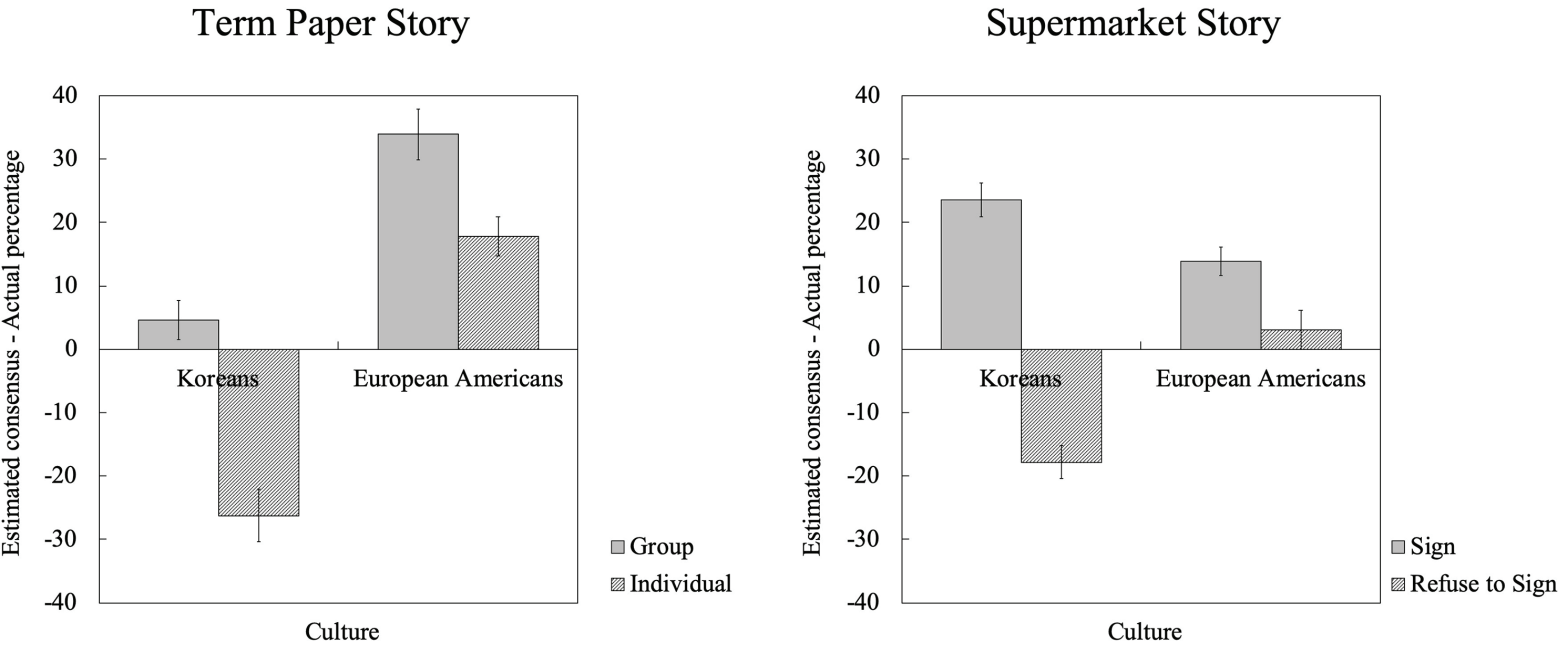
Accuracy: Actual percentage of people who chose option 1 (voting for group papers in the Term Paper story and signing the release in the Supermarket story) was subtracted from the estimated percentage for option 1 by culture and raters’ personal choices.

However, this pattern was further qualified by a significant interaction effect between Culture and Rater’s Choice [Paper: *F*(1, 97) = 4.18, *p* < 0.05, $\eta_{p}^{2}$ = 0.041; Supermarket: *F*(1, 97) = 33.67, *p* < 0.001, $\eta_{p}^{2}$ = 0.258]. The significant interaction effect seemed to be driven by Korean’s tendency to underestimate the consensus for the option they did not choose as well as to overestimate the consensus for the option they choose (see Figure 2). Koreans who chose option 1 overestimated the percentage of the people who would choose the same option, which is indicated by the positive values of the different scores [Paper: *t*(30) = 1.87, *p* < 0.080; Supermarket: *t*(23) = 9.67, *p* < 0.001]; but most importantly, Koreans who actually chose option 2 significantly underestimated the percentage of the people who would choose option 1, which is indicated by the significant negative values of the difference scores when compared with the value of zero [Paper: *t*(17) = −6.73, *p* < 0.001; Supermarket: *t*(24) = −6.36, *p* < 0.001]. This result suggests that the cultural difference found in this study was mainly driven by Koreans’ tendency to perceive oneself to be the “ordinary” by overestimating the consensus for the choice they made and by underestimating the consensus for the choice they did not make.

In sum, this study revealed the existence of a cultural difference in the FCE: Koreans were found to show a greater FCE than Americans in a task involving personal opinion in hypothetical situations. In addition, our analysis on the accuracy of estimation with difference scores revealed that the cultural difference found in this study was primarily driven by Koreans’ tendency to overestimate the consensus for the choice they made and to underestimate the consensus for the choice they did not make. It seems that overestimating the consensus for the choice they made was not enough for Koreans; they may have needed to go even further to underestimate the consensus for the other option in order to feel that they are safe in the ordinary.

Still, some might raise the possibility that the scenarios and behavioral choices we examined in this study were culture-bound. For example, in the Term Paper story, East Asians are likely to prefer group papers, whereas Westerners prefer individual papers; and this difference in the prevalence of choice for each story might complicate the cultural comparison, and even more, be the source of the cultural pattern of the FCE.

Koreans, in fact, were more likely to opt for the group option than Americans: 63% (63.27%) of Korean participants opted the group paper option, whereas only 35% (35.29%) of American participants opted the same in our sample (Table 3). However, the response rate for the Supermarket story shows exactly the opposite cultural pattern: a majority (65.38%) of American participants opted to sign the release; however, only 48.98% of Korean participants opted the same. Still, the cultural pattern of the FCE (perceived consensus, Figure 1) was parallel across the two stories. In addition, the additional accuracy analysis with difference scores, which adjusts the estimated consensus with the actual consensus from each culture, shows that the pattern of accuracy (Figure 2) is also parallel across the two stories. This suggests that the cultural difference in the FCE goes beyond the specific cultural preferences (e.g., Koreans preferring option 1 while Americans preferring option 2) tied to a scenario.

One of the contributions of this study lies in that our analysis on the accuracy of the FCE provided a unique opportunity to examine two competing hypotheses regarding the direction of the potential cultural difference. As reviewed earlier, a substantial body of cultural literature acknowledges the fact that people from East Asian culture, characterized by collectivism, an interdependent self and a social goal of harmony, are more sensitive to consensus information and norm and perceive themselves as similar to others than their Western counterpart. This line of research predicted that Koreans should show stronger FCE than European Americans. However, other studies focusing on the fact that East Asians tend to pay attention to others and are generally more accurate in estimating the prevalence of others’ behaviors predicted the very opposite: East Asians might succumb less to FCE compared to Westerners.

Our results from the accuracy analysis indicated that there is no evidence that Koreans are more accurate than European Americans in estimating the consensus of opinions in general. Furthermore, their accuracy in an absolute sense did not seem to contribute to the cultural pattern we found for the FCE. Koreans did seem to have better knowledge of others and show some level of accuracy in certain contexts (e.g., Koreans who chose option 1 in the Term Paper Story); however, this cognitive advantage did not override the estimation process to yield less FCE in Koreans.

## General Discussion

This research examined the potential cultural variations of the FCE and attempted to fill the gap in the literature. By examining the cultural effects in the original settings that were used in the initial study on FCE by Ross et al. (1977), this study attempted to provide a proper base for a cross-cultural comparison and establish the cultural pattern. The two studies comparing the FCE between two cultures demonstrated both universal and cultural specificity: This research confirmed the general existence of the tendency that people overestimate the prevalence of their own choices across cultures; however, a more important contribution comes from the fact that this study revealed some noticeable cultural variations that had been anticipated.

First, the examination of the choice domains revealed some cross-cultural pattern. Findings from Study 1 demonstrated that cultural variation existed in the type of items that was more susceptible to the effect. For example, people from both cultures demonstrated stable FCEs in items regarding Political Expectations and Personal Problems, and Koreans were found to demonstrate greater FCE than US Americans in these domains.

However, the items related to Personal Traits and Views revealed a different pattern: Koreans did not show stronger FCE than US Americans. Additional analysis demonstrated that unlike US Americans, Koreans’ estimation on others’ traits and views generally did not deviate from the point of 50, in which the pattern was not observed in the estimation on other domains, i.e., Political Expectations or Personal Problems. This finding supports the notion on the difference of self-clarity (i.e., the consistence of the self) between East Asians and North Americans (e.g., Heine, 2001). While North Americans’ independent self values the abstract traits and inner properties consistent across situations as a representation of their individuality, it is not the case for East Asians with an interdependent self, which considers roles and relationships, rather than stable inner traits, to be more important.

This result might seem to contradict to Park’s (2012) finding, which states that the FCE for attribute items was greater among Koreans than among Americans; however, a closer look at the study reveals that it is not the case. The three attribute items that were used in the Park’s (2012) study was rather specific behaviors such as “taking good notes,” “promptly returning things or money borrowed from other people,” and “showing up on time when meeting other people,” which is different from the personality traits that we used in the study, which defines broad sense of the self. Furthermore, the author also noted one of the reasons that might lead to the greater FCE in these items for Koreans lies in the fact that the attribute items used in the study pertain to social relationship maintenance features, indicative of concern for others, and group harmony, which does not apply to our study.

The various strengths and patterns of the effect across item categories between two cultures suggest that decisions and choices regarding different items might be subjected to different motivational forces and cognitive advantages/disadvantages. It might be possible that item categories demonstrating certain patterns of the FCE point to the location of motivational or cognitive importance of each culture. Likely, differential needs and focuses specific to each culture might have determined “where” people show more pronounced FCE. This possibility awaits further scrutiny.

Furthermore, we were able to find cultural differences in the FCE in terms of the relative size of the effect. Overall, Koreans seem to show stronger FCE than European Americans, especially regarding personal choices involving political expectations and personal problems (Study 1) and behavioral choices in hypothetical situations (Study 2).

This result seems to be consistent with what previous literature on cultural studies has suggested in the light of the traditional accounts of the FCE. Two major accounts that provide explanations for the FCE are *selective exposure* and *causal focus*. *Selective exposure* or availability account of the FCE focuses on the fact that people tend to know and associate with others with whom they share their background, experiences, and interests; furthermore, it maintains that this selective exposure to this similar others and the availability of information about them breeds a false consensus (Sherman et al., 1983). In a collectivistic and interdependent culture, such as in East Asian culture that values group identities and the goal of social harmony (e.g., Kim and Markus, 1999), it is very likely that people not only seek and perceive more similarity in others who may be potential candidates for future interaction, but also they in fact interact with more like-minded people. These observations bolster the notion that East Asians are selectively exposed to similar others more than Westerners, which can explain the stronger FCE among Koreans than among European Americans, whose culture value is unique.

*Causal focus* account of the FCE, suggested by Gilovich et al. (1983), also adds support for the direction of the cultural variation. This account maintains that people’s causal analysis of their attitudes and behaviors is one determinant of the false consensus effect. For example, if causal analysis focuses on situational aspects, individuals will infer that the powerful situational influence should govern the behavior of others as well as their own, and the false consensus should be strengthened. Well-documented findings now confirm that people in Eastern cultures tend to accept the power of situational influence more than their Western counterpart and thus be less susceptible to the fundamental attribution error (e.g., Miller, 1984; Morris and Peng, 1994; Lee et al., 1996). In this sense, it is not surprising to find that East Asians extend the commonness of their own choices to a greater degree than Westerners. Together, these accounts seem to support the tendency of East Asians demonstrating stronger FCE than Westerners.

In addition, our findings negated the alternative prediction that East Asians might succumb less to the FCE based on the fact that they tend to have better knowledge of others than European Americans. However, it is possible that East Asians have relatively more accurate knowledge of others than their Western counterparts; however, results from our studies suggested that this particular cognitive advantage did not completely override other biases that might be responsible for the FCE in East Asians.

Our analysis on the accuracy of estimation in Study 2 seems to offer partial support for this point. Koreans who chose the group paper (Option 1) in the Term Paper story seemed to estimate the consensus pretty accurately. However, Koreans who chose the individual paper (Option 2) underestimated the percentage of people who endorsed the other option, which led to greater FCE than European Americans. It strongly suggests that there is something more than better knowledge of others or need for accuracy that drives the specific pattern in Koreans.

Our analysis on accuracy with difference scores between the estimated and actual consensus revealed that stronger FCE on the part of South Koreans is in part due to their tendency to overestimate the prevalence of people who chose the same option to their own and to underestimate the prevalence of people who chose an option other than their own. Unlike independent North Americans who emphasize the value of uniqueness, interdependent East Asians are known to value modesty and harmony (Markus and Kitayama, 1991). In addition, it is suggested that East Asians may have a strong motivation to see oneself as normal and ordinary and hold a belief that being ordinary is safe and also beneficial (e.g., Ohashi and Yamaguchi, 2004). Overestimating the consensus on one’s own position is one way to acquire the sense of “ordinariness”; however, underestimating the consensus of alternative position can be another way to achieve it, and this pattern seems to have contributed to a greater FCE in Koreans. Whether Koreans or East Asians, in general, have stronger motivation to see themselves as ordinary and whether this can be achieved through the underestimation of alternative choice as well as overestimation of one’s own choice is a research question that requires further inquiry.

In sum, this research examined the potential cultural variations of the FCE and attempted to fill the gap in the literature. It was found that generally, East Asians tended to overestimate the prevalence of their choices to a greater degree than Westerners, and this cultural pattern varied depending on the domains of choices. Previous literature allowed us to speculate on the possible reasons for the observed cultural variations, and these accounts seem to provide plausible explanations for the cultural patterns. However, the design of this research did not allow us to determine which processes were exactly responsible for the cultural patterns. Examining the exact mechanism underlying this cultural pattern requires to experimentally manipulate some of the factors, either cognitive or motivational, or to include additional measures to specifically look into how people in each culture perceived the similarity or social distance of the target group or construed the alternatives. This awaits further scrutiny.

Since we initially had little evidence to work on to discuss the potential cultural variations of the FCE, we attempted to establish the phenomenon by using the original settings of Ross et al.’s (1977) initial study. This approach was beneficial in that we could establish the existence of the cultural difference in the FCE by using well-established task settings and compare the results with the original study to clarify the variations and similarities. However, this replication approach also imposed some restrictions on our investigation. For example, their choice of categories and related items for the FCE, which we adopted for our Study 1, was rather arbitrary and was not theory-driven. This setting limited our opportunities to investigate more specific predictions regarding the cultural differences. In addition, their original setting was not designed for the cross-cultural comparison, which makes the simple comparison for some of the original items problematic. Although replicating Ross et al.’s (1977) original study provided a good starting point to examine and discuss the cultural variations of the FCE and offered a vantage point for cultural variations that have not been fully discussed, it asks for a future research with more theory-driven predictions based on the cultural literature.

From a motivational account perspective, the FCE was construed as a consequence of justifying one’s own behavioral choices to be appropriate and also as rational responses to the demands of the environment, and therefore, the FCE was conceived as an “egocentric” bias. Partly due to its labeling, it was generally theorized that egocentric biases such as this one should be less evident in collectivist cultures in which consideration of others is more emphasized (e.g., Gilovich et al., 1999). East Asians, typically characterized as “collectivistic” or “interdependent,” were sometimes portrayed to be far more caring, considerate, and understanding compared to Westerners, which in turn have led people to think them to be less susceptible to “egocentric” biases. However, our findings suggested otherwise: Koreans too are subject to a seemingly egocentric bias, such as the FCE, even more than European Americans. This result resonates with other researchers’ observations pointing out that a strong commitment to social sensitivity and perspective taking does not necessarily lead to a better understanding of others (Davis and Kraus, 1997; Vorauer and Cameron, 2002).

This observation leads us to a thought that even for East Asians who are reputed to be “social experts,” there might be some ignored social blind spots that might have important implications for decision making and negotiation across cultures. For example, a tendency to assume or perceive greater consensus for one’s choices may lead to overconfidence in one’s behaviors and decisions. In fact, previous research has reported that people in East Asian culture also showed a greater overconfidence effect than Westerners (Wright et al., 1978; Yates et al., 1989, 1996; Lee et al., 1995; Whitcomb et al., 1995). In negotiation settings, being overconfident was found to decrease the negotiators’ willingness to make concessions (Neale and Bazerman, 1985).

Furthermore, it seems that some characteristics of East Asian culture can be combined to place East Asians at a particularly higher risk of misunderstanding. For example, East Asian culture is generally characterized as a high context society built on “assumed” common ground and agreement that will not be communicated in a direct and concrete manner. Likely, saving face is considered as important in this culture, and in some contexts, the need to save face can also prevent people from communicating one’s opinions and decisions. These aspects in East Asian culture can easily remove opportunities to share one’s opinion with others or verify the social reality. Without deliberate and exquisite attempts to understand others and communicate one’s position, these societies can easily fall into a curse of knowledge and might not be able to achieve the optimal social decisions based on the “real” consensus. Therefore, future research should look into the consequences of perceiving false consensus, particularly in East Asian culture, and their implications for cross-cultural communication and negotiation. In a sense, a surge of cultural studies seems to have led to the idealization of cultures to some degree; it sometimes obscures the real nature of their social realities and its consequences. The findings from this research suggest that a more cautious approach for making assumptions for different cultures could be both useful and beneficial.

## Ethics Statement

The research reported in this manuscript adhered to ethical guidelines and legal requirements of the study countries. The study involved providing estimates regarding mostly hypothetical situations, and participants were informed that they can quit at any time if they want. All participants provided written informed consent.

## Author Contributions

IC contributed to conception and design of the study. IC organized and supervised the collection and the analyses of data. OC performed the statistical analyses and wrote the first draft of the manuscript. Both authors contributed to proofread and revise the manuscript and read and approved the submitted version.

### Conflict of Interest

The authors declare that the research was conducted in the absence of any commercial or financial relationships that could be construed as a potential conflict of interest.

## Footnotes

^1^Analysis on individual items showed that Koreans’ estimates on most of the items were not different from 50 [Shy: *t*(53) = −1.01, *p* = 0.32; Optimistic: *t*(53) = 1.06, *p* = 0.29; Competitive: *t*(53) = 4.49, *p* < 0.001; Support of women’s liberty: *t*(53) = −1.60, *p* = 0.12], whereas those provided by European Americans were significantly different from 50 [Shy: *t*(68) = −6.13, *p* < 0.001; Optimistic: *t*(69) = 7.19, *p* < 0.001; Competitive: *t*(69) = 10.48, *p* < 0.001; Support of women’s liberty: *t*(69) = 8.69, *p* < 0.001].

^2^Analysis on individual items showed that estimates from both Koreans [Women president: *t*(53) = −10.69, *p* < 0.001; poverty: *t*(53) = −4.86, *p* < 0.001; extraterrestrial life: *t*(53) = −5.20, *p* < 0.001; nuclear warfare: *t*(53) = −9.98, *p* < 0.001] and Americans [Women president: *t*(68) = −6.71, *p* < 0.001; poverty: *t*(68) = −3.82, *p* < 0.001; extraterrestrial life: *t*(68) = −6.61, *p* < 0.001; nuclear warfare: *t*(67) = −3.82, *p* < 0.001] were significantly different from 50.

^3^Analysis on individual items showed that estimates on personal problems for both Koreans [thinking about dying: *t*(53) = 9.75, *p* < 0.001; making friends: *t*(53) = −2.92, *p* < 0.01; controlling temper: *t*(53) = −2.29, *p* < 0.05; depressed: *t*(53) = −1.75, *p* = 0.09; satisfying emotional needs: *t*(53) = −2.06, *p* < 0.05] and Americans [thinking about dying: *t*(68) = −3.42, *p* < 0.01; making friends: *t*(68) = −11.98, *p* < 0.001; controlling temper: *t*(68) = −5.16, *p* < 0.001; depressed: *t*(68) = −9.73, *p* < 0.001; satisfying emotional needs: *t*(67) = −1.17, *p* = 0.25] were significantly different from 50 and usually less than 50. However, estimates on one item, i.e., thinking about dying, provided by Koreans were significantly larger than 50, which deviated the general pattern and yielded a non-significant result overall for Koreans [Koreans: *t*(269) = 0.15, n.s.; Americans: *t*(343) = −10.32, *p* < 0.001]. This seems to reflect the fact that suicide is the fifth major cause of death in Korea (Statistics Korea, 2019) and Korea’s suicide rate is the highest among the OECD countries (OECD, 2019). When we excluded the item on thinking about dying from the analysis, estimates from both Koreans and Americans were significantly different from 50, and this result is reported above.
